# Cement pulmonary embolism as a complication of percutaneous vertebroplasty in cancer patients

**DOI:** 10.1186/s40644-018-0138-8

**Published:** 2018-02-08

**Authors:** Asem Mansour, Nayef Abdel-Razeq, Hussein Abuali, Mohammad Makoseh, Nouran Shaikh-Salem, Kamelah Abushalha, Samer Salah

**Affiliations:** 1 Department of Radiology, King Hussein Cancer Center, Amman, Jordan; 2 Istishari Hospital, Amman, Jordan; 3 Department of Internal Medicine, King Hussein Cancer Center, Amman, Jordan

**Keywords:** Vertebroplasty, Cement, Pulmonary embolism, Cancer, Osteoporosis

## Abstract

**Background:**

Vertebroplasty is a minimally invasive procedure commonly performed for vertebral compression fractures secondary to osteoporosis or malignancy. Leakage of bone cement into the paravertebral venous system and cement pulmonary embolism (cPE) are well described, mostly in patients with osteoporosis. Little is known about the clinical sequelae and outcomes in cancer patients. In this study, we report our experience with cPE following vertebroplasty performed in cancer patients.

**Methods:**

Records of all consecutive cancer patients who underwent vertebroplasty at our institution were retrospectively reviewed. The procedure was performed via percutaneous injection of barium-opacified polymethyl-methacrylate cement.

**Results:**

A total of 102 cancer patients with a median age of 53 (19–83) years were included. Seventy-eight (76.5%) patients had malignant vertebral fractures, and 24 (23.5%) patients had osteoporotic fractures. Cement PE was detected in 13 (12.7%) patients; 10 (76.9%) patients had malignant fractures, and the remaining three had osteoporotic fractures. Cement PE was mostly asymptomatic; however, 5 (38.5%) patients had respiratory symptoms that led to the diagnosis. Only the five symptomatic patients were anticoagulated.

Cement PE was more common with multiple myeloma (MM); it occurred in 7 (18.9%) of the 37 patients with MM compared with only three (7.3%) of the 41 patients with other malignancies. No difference in incidence was observed between patients with osteoporotic or malignant vertebral fractures.

**Conclusions:**

Cement PE is a relatively common complication following vertebroplasty and is mostly asymptomatic. Multiple myeloma is associated with the highest risk. Large-scale prospective studies can help identify risk factors and clinical outcomes and could lead to better prevention and therapeutic strategies.

## Background

Vertebral body compression fractures are common, especially among elderly patients. The high prevalence of osteoporosis and cancer in this age group is a major contributing factor [1–3].

In addition to severe pain requiring hospital admission and parenteral opioids, these fractures can cause neurological deficits, height loss and kyphosis with associated restrictive pulmonary disease [4, 5].

Vertebroplasty is a minimally invasive procedure that is commonly performed for vertebral compression fractures secondary to both osteoporosis and malignancy [6]. The procedure is performed under image guidance and involves the injection of a cement polymer, commonly polymethylmethacrylate (PMMA), into the vertebral body to confer improved stability and pain relief [7, 8]. Vertebroplasty was first introduced at the University Hospital of Amiens, France, in 1984, when it was used to augment the post-resection defect of a benign spinal tumor [9]. Since then, it has become an increasingly recommended therapeutic intervention due to its high efficacy and safety [10].

**Fig. 1:**
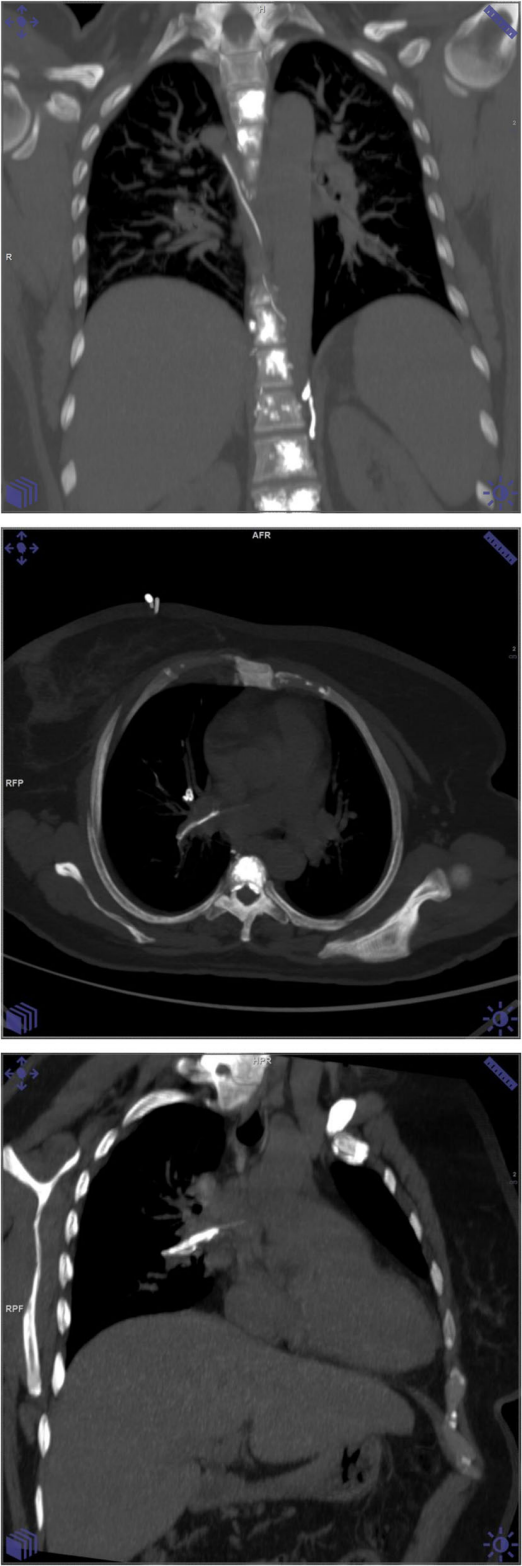
Selected coronal image with maximum intensity projection of unenhanced chest CT showing multilevel vertebral augmentation by cement. There is a branching linear density along the course of the lumber veins and the azygus/hemiazygus system representing cement leak/ intravasation. Axial and coronal oblique MIP images are showing a similar linear density in the right main pulmonary artery extending to the right superior segmental branch of the right lower lobe representing cement pulmonary emboli

Vertebral bodies are highly vascularized and form a valveless network with the paravertebral and extradural venous plexuses. Vertebral compression fractures enhance venous drainage and facilitate migration of cement fragments into the systemic venous circulation. Leakage of bone cement into the paravertebral venous system is well described (Fig. 1) [11]. Cement pulmonary embolism (cPE) is a well-recognized complication of vertebroplasty in patients with traumatic, osteoporotic, and metastasis-induced compression fractures [12–14]. The clinical implications and complications associated with such embolisms, especially in cancer patients, are poorly characterized.

In this study, we report our experience with vertebroplasty performed for vertebral fractures in cancer patients. We also provide a literature review of published data regarding cPE in cancer and non-cancer patients.

## Methods

This is a retrospective study in which the hospital’s and the radiology department’s databases were searched for both “vertebroplasty” and “cPE”. The records of all patients who underwent vertebroplasty at our institution over the past ten years were reviewed. All imaging studies including computed tomography (CT) scans or plain chest radiographs that were performed following vertebroplasty for any indication were reviewed again by an experienced radiologist to assess for features of cPE.

The following data were extracted from patients’ charts and electronic medical records: gender, age at time of vertebroplasty, type and stage of cancer, number and location of vertebral metastases, status of the patient before vertebroplasty (ambulatory or hospitalized), and history of cardiac or pulmonary diseases diagnosed before the vertebroplasty. Moreover, we gathered data pertaining to imaging tools that were utilized for diagnosis of cPE, clinical manifestations, details of treatment, hemodynamic sequelae associated with cPE, and dates of last follow-up and death.

The procedure was performed under image guidance via a percutaneous injection of barium-opacified polymethyl-methacrylate cement.

Given its retrospective nature, our research was exempted from review by our Institutional Review Board (IRB).

## Results

A total of 102 patients underwent vertebroplasty during the study period and were included in this report. All had a pathology-confirmed diagnosis of cancer. The median age was 53 years (range: 19–83), and 57 (55.9%) patients were female.

Pathological evaluation of the fractured vertebrae confirmed metastatic disease involvement in 78 (76.5%) patients. The fractures in the other 24 (23.5%) patients, many with active cancer, were due to osteoporosis. Vertebroplasty was performed in the lumbar spine in 57 (55.9%) patients and in the thoracic spine in 16 (15.7%) patients; 29 (28.4%) patients had the procedure performed in both lumbar and thoracic vertebrae.

Multiple myeloma was the commonest primary cancer and was identified in 37 (36.3%) patients, followed by breast cancer in 25 (24.5%) patients and lymphoma in 13 (12.7%) patients. Most patients (95.1%) were ambulatory at time of vertebroplasty; only 5 were wheelchair-bound. In addition to cancer, many patients had other comorbidities, including significant cardiac illness in 19 (18.6%) patients and significant pulmonary disease in 9 (8.8%) patients had (Table 1).

**Table 1:** Patients’ characteristics

Age	
Median (years)	53
Range (years)	19–83
Sex	
Male	45 (44.1%)
Female	57 (55.9%)
Smoking history:	
Smoker	17 (16.7%)
Ex-smoker	18 (17.6%)
Never smoked	67 (65.7%)
Underlying cancer:	
Multiple Myeloma	37 (36.3%)
Breast	25 (24.5%)
Lymphoma	13 (12.7%)
Lung	5 (4.9%)
Sarcoma	4 (3.9%)
Prostate	4 (3.9%)
Others	14 (13.7%)
Timing of vertebral fracture:	
Initial presentation	57 (55.9%)
Disease recurrence	45 (44.1%)
Comorbidities:	
Hypertension	32 (31.4%)
Diabetes mellitus	26 (25.5%)
Cardiac disease	19 (18.6%)
Pulmonary disease	9 (8.8%)
Mobility:	
Ambulatory	97 (95.1%)
Wheel-chair bound	5 (4.9%)
Underlying etiology and site:	
No metastasis (Osteoporosis)	24 (23.5%)
Vertebral metastasis^a^:	78 (76.5%)
Cervical spines	61 (78.2%)
Dorsal spines	73 (93.6%)
Lumbar spines	75 (96.2%)

^a^Total > 102, reflecting multiple sites

**Fig. 2:**
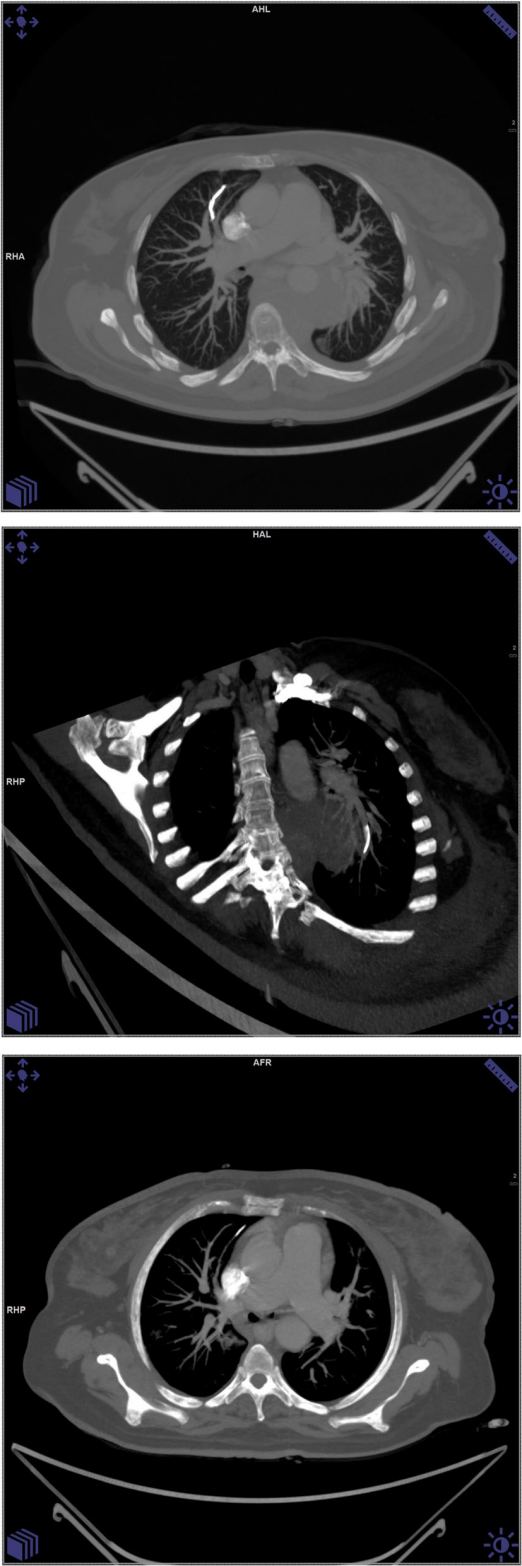
Chest CT with MIP showing a curvilinear density in the anterior subsegmental branch of Right Upper lobe indicating cement PE. Oblique coronal MIP image of the same patient showing cement PE in basal subsegmental branch of LLL

Cement PE was identified in 13 (12.7%) cases; the median age of patients with cPE was 50 years (range: 31–81). Eleven (84.6%) patients were diagnosed by CT scan (Fig. 2), while the other two patients had an initial suspicious finding on chest X-ray that was confirmed by a follow-up CT. All patients with confirmed cPE except one were ambulatory at the time of vertebroplasty.

The total number of levels injected was 252 (mean per patient: 2.47, median: 2). As such, the incidence of cPE is 5.2 episodes per 100 injections. Among the 13 patients with cPE, a total of 39 levels were injected (mean: 3.0, median: 3).

Cement PE was mostly asymptomatic, but 5 (38.5%) patients had symptoms (dyspnea, chest pain, cough, tachycardia, hypoxia) that led to the diagnosis. Only the five symptomatic patients were anticoagulated; all were treated with low molecular weight heparin (LMWH) and none had any minor or major bleeding or any other complication related to anticoagulation. Ten (76.9%) of the 13 patients had malignant vertebral fractures, while the other three (23.1%) patients had osteoporotic fractures with no known vertebral metastasis. Details of the 13 patients are listed in Table 2.

**Table 2:** Characteristics and outcome of patients with cement pulmonary embolism

Number	Age	Gender	Underlying Etiology^b^	Primary Cancer	Location	Type of PE	Symptoms	Anticoagulation	Status	Survival (Months)
1	31	F	Malignant	Breast	T12-L2	Main	Dyspnea, Tachycardia, Hypoxia	No	Dead	8.5
2	41	F	Malignant	Breast	L1-L5	segmental	Dyspnea, Tachycardia, Hypoxia	LMWH	Dead	1.5
3	78	F	Osteoporosis	Lymphoma	L1	segmental	No	No	Alive	53
4	55	M	Osteoporosis	Lung	L2- L5	Lobar	No	No	Dead	12
5	81	F	Osteoporosis	Lymphoma	L5	subsegmental	No	LMWH	Alive	48
6	37	M	Malignant	MM	T9–12,L1	subsegmental	No	No	Dead	NA^a^
7	50	F	Malignant	MM	T5-T8	subsegmental	No	No	Dead	24
8	63	M	Malignant	MM	L1-L3	Lobar	No	No	Dead	20
9	50	M	Malignant	MM	L2-L4	subsegmental	No	No	Alive	48
10	68	M	Malignant	MM	T12,L1,L2	subsegmental	Dyspnea, Chest pain, Cough, Tachycardia, Hypoxia	No	Dead	4.5
11	60	F	Malignant	Breast	L4	subsegmental	No	Already on LMWH	Dead	11
12	45	F	Malignant	MM	L3-L5	Main	Dyspnea	LMWH	Dead	8
13	45	F	Malignant	MM	T12,L1,L2	Lobar	Dyspnea	LMWH	Dead	8

*LMWH* Low molecular weight heparin, *MM* Multiple Myeloma

^a^NA: Data not available, ^b^all in cancer patients

Cement PE was more common among patients with MM compared to other malignancies; it occurred in seven of 37 (18.9%) MM patients and 3 of 41 patients (7.3%) with other malignancies (HR = 0.338; 95% CI, 0.081–1.42; *P =* 0.18).

The nature and etiology of vertebral fractures are also worth addressing. In all seven myeloma patients with cPE, the fracture was related to malignant tumor infiltration but the two lymphoma cases were related to osteoporosis and not malignancy; both lymphoma cases were in elderly women (78 and 81 years old) who had received a significant amount of steroids as part of their lymphoma treatment.

The incidence of cPE was not different in patients with osteoporotic (3/24, 12.5%) or malignant (10/78, 12.8%) vertebral fractures (HR = 0.972; 95% CI, 0.244–3.86; *P* = 0.99). However, patients with osteoporotic vertebral fractures were older, with a median age of 66 years compared to 51 years for patients with malignant fractures (*P* = 0.0076).

Survival following the vertebroplasty and associated cPE was variable; one patient survived only 6 weeks, while 3 others are alive at 48 months or more. Death was not attributed to PE in any of the patients.

## Discussion

Several studies and case reports have addressed the issue of cement leakage and cPE in patients undergoing vertebroplasty, with conflicting conclusions.

Our literature search retrieved many papers addressing this topic; the majority consisted of case reports, mostly in non-cancer patients. However, some papers include a few cancer cases in series with a majority of non-cancer patients [12–14].

The reported frequency of cPE was variable. Variation in imaging modalities, screening strategies and patient populations studied contributed to this marked variation [15, 16]. In our study, the small number of patients with cPE makes it difficult to correlate the risk of cPE to the number of injections performed. Other variables including site, severity of resulting deformity, body mass index (BMI), primary tumor and additional comorbidities may contribute.

In one study, chest radiographs were available in 64 of 69 percutaneous vertebroplasty procedures and, upon retrospective review, cPE was noted in only three (4.6%) cases [17]. In another study, CT scans of the chest were obtained routinely and cPE was identified in 18 (23%) of 78 procedures performed in patients with osteoporotic, non-malignant fractures [18].

In a more recent study, VERTOS II, chest CT was obtained for all patients post-vertebroplasty regardless of symptoms; 14 (26%) of 54 patients had cPE and all were asymptomatic [19].

Data addressing the clinical manifestations and risk factors for cement pulmonary emboli in cancer patients are very limited, as much of the published literature involves patients with osteoporotic fractures. A few studies include small numbers of cancer patients among larger series of patients with vertebral fractures related to osteoporosis and not cancer [18–20].

Although data from the literature suggest that most patients with cPE as a complication of osteoporotic compression fractures were diagnosed through screening radiographic studies and remained free of symptoms or long-term adverse pulmonary sequelae [16], five (38.5%) of our 13 patients had symptoms (dyspnea, cough, chest pain, tachycardia, hypoxia) that led to the diagnosis. While it is true that such respiratory symptoms in patients with cancer can be attributed to a variety of pulmonary disorders (lung metastasis, pleural effusions, smoking-related chronic obstructive pulmonary airway disease (COPD) or even thrombotic PE), none of the five symptomatic patients in our study had any other pathology that could explain their symptoms.

While there is no standard of care for the treatment and management of cPE, asymptomatic individuals may be effectively managed conservatively with close clinical monitoring [21]. This conclusion is supported by the fact that none of the asymptomatic patients in our series had any clinical sequelae. However, all five symptomatic patients were fully anticoagulated. Anticoagulation can help prevent progressive pulmonary artery occlusion [22].

The increasing use of vertebroplasty and the incidence of associated symptomatic and asymptomatic cPE highlight the importance of a better understanding of this commonly encountered complication. Adequate efforts should be made to identify cancer patients who are at risk of this complication and to identify appropriate screening and therapeutic strategies.

Many questions remain unanswered. Should we recommend that routine imaging studies be performed after vertebroplasty [23]? Should we anticoagulate all asymptomatic patients diagnosed by routine imaging studies performed after the procedure? Is a location (cervical, thoracic, or lumbar vertebrae) associated with higher risk? What is the natural history of cPE? Further research is needed to address these knowledge gaps regarding cPE in cancer patients.

## Conclusions

Cement PE, while mostly asymptomatic, is a relatively common complication following vertebroplasty. Multiple myeloma is the commonest malignancy associated with this complication. Large-scale prospective studies can help identify risk factors and clinical outcomes, and help develop better prevention and therapeutic strategies.
